# The screening for chronic kidney disease among older people across Europe (SCOPE) project: findings from cross-sectional analysis

**DOI:** 10.1186/s12877-020-01701-w

**Published:** 2020-10-02

**Authors:** Andrea Corsonello, Ellen Freiberger, Fabrizia Lattanzio

**Affiliations:** 1 Italian National Research Center on Aging (IRCCS INRCA), Ancona, Fermo and Cosenza, Cosenza, Italy; 2 Department of Internal Medicine-Geriatrics, Institute for Biomedicine of Aging (IBA), Friedrich-Alexander-Universität Erlangen-Nürnberg, Erlangen, Germany

Editorial

The scenario of CKD in the older population is known to be very complex. Among community dwelling older people there is a broad range of different functional levels ranging from fitness and robustness to frailty or disability. Impaired physical function, frailty and disability, cognitive impairment, depression, vision and hearing impairment, malnutrition, and sarcopenia all contribute to worsen health outcomes and to increase the use of health care resources, thus challenging health care systems. The benefits of preventing/slowing the progression of CKD among these patients have the potential to impact different social and health domains, e.g. reducing the need for long-term care and the cost related to caregiving. Thus, early identification of CKD represents a relevant issue to be addressed in the older population.

At the end of 2013 the American College of Physicians (ACP) issued a statement against the usefulness of chronic kidney disease (CKD) screening for the general adult population [1]. Those recommendations were severely criticized by nephrologists, fully aware that early detection and intervention can slow the progression of CKD to renal failure. Noting the lack of involvement of nephrologists in the development of ACP statements, Molitoris claimed that “*We should be committed to supporting all approaches that will change kidney disease from a silent disease too often discovered in its late stages to one that is identified when modifications can affect the progression of the disease [ … ]. The entire medical community must act sooner and enlist patients’ help in minimizing the comorbidities and progression that too often remain undetected until very late in the disease process”* [2].

CKD impact on global health, either as a risk factor for morbidity and mortality or by causing cardiovascular disease. A recent systematic analysis of the Global Burden of Disease Study 1990–2017 [3] showed that over the past 27 years CKD burden has not declined to the same extent as many other important non-communicable diseases. However, CKD and its consequences remain largely preventable and treatable and deserves greater attention in global health policy decision making. Indeed, several studies suggest that screening for CKD in high-risk and older populations may represent a cost-effective approach to reduce progression to renal failure and CKD mortality [4–7].

Current screening measures rely on creatinine-based estimated glomerular filtration rate (eGFR). They are known to have some degree of inaccuracy when used in older people and to carry an increased risk of both over- and under-diagnosis, but alternative filtration markers have been identified and are worth of testing. Indeed, a screening program should take into account the characteristics of the target population to achieve effective results. A high proportion of older CKD patients are usually affected by multimorbidity, polypharmacy, frailty, functional and cognitive impairment and disability [8–16]. However, CKD screening programs to date have not included the use of the comprehensive geriatric assessment (CGA), the only assessment technology able to capture the numerous dimensions of health status and their complex interactions in older people. Accordingly, comparing different CKD screening methods for older complex patients in regards to their accuracy is considered a very important issue to be addressed. Indeed, this view is widely shared by the geriatric and nephrologist communities [17, 18], and the implementation of innovative findings is expected to improve CKD screening and management in such a vulnerable population (Fig. 1).

**Fig. 1:**
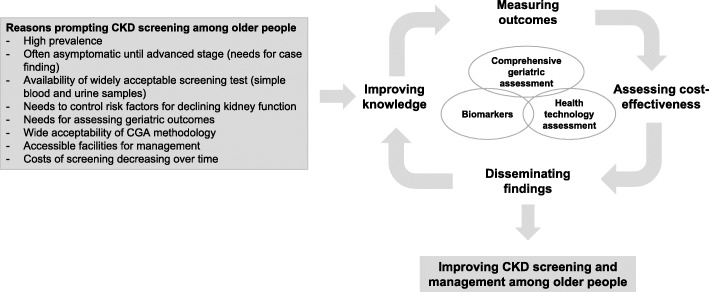
Pathways from comprehensive screening to improvement of CKD management among older people

All these aspects have been the primary motivator for the development of a EU funded research project, titled “Screening for CKD among Older People across Europe (SCOPE)”, aiming at comparing different screening methods for CKD in the older population, including a comprehensive set of CGA variables to explore population-specific outcomes [19].

Initial results from the baseline database of the SCOPE project [19] have been made available for the participating researchers in this special issue as a first description of the population studied, a cohort of 2464 patients from 7 countries, through a series of cross-sectional analyses about typically geriatric topics.

The main finding from the study by Moreno-Gonzalez et al. is the relevant prevalence of sarcopenia observed among community-dwelling people aged 75 years and older, using the most recent diagnostic criteria for sarcopenia endorsed by the EWGSOP2 consensus. Participants within poorer eGFR categories, irrespective of the equation used for its calculation, and albuminuria have a higher prevalence of sarcopenia and are more often severely sarcopenic. However, there are some differences in prevalence according to the eGFR formula used.

In the paper by Guligowska et al., the SCOPE study population shows generally features of overweight and obesity with small prevalence of malnutrition. Higher weight, circumferences, body mass index (BMI) and waist-to-height ratio (WHtR) were significantly associated with increased likelihood of having CKD, whilst higher Mini Nutritional Assessment (MNA) score and higher serum albumin were inversely associated with CKD. The risk of MNA < 24 or hypoalbuminemia was increased among patients with estimated glomerular filtration rate (eGFR) < 30 as compared to those with eGFR> 60 ml/min/1.73 m^2^.

Corsonello et al. showed that CKD contributes significantly to multimorbidity patterns in a population of older outpatients and it was rarely observed without any co-occuring disease. The most significant co-occuring pairs involving CKD included hypertension, anemia, CHF, atrial fibrillation, myocardial infarction, hip fracture, and to a lesser extent hearing impairment, diabetes and cancer. Cluster analysis showed that CKD may cluster with hypertension and sensory impairments. CKD severity and physical performance as assessed by the Short Physical Performance Battery (SPPB) test may be associated with not negligible changes in both co-occuring pairs and multimorbidity clusters. These findings strengthen the need of including disease severity in multimorbidity studies. Additionally, assessing physical function and investigating interventions targeting physical function among complex multimorbid patients represents a research priority to improve outcomes and reduce costs associated with multimorbidity.

Britting et al. showed that CKD, at least in the early stages, is not a predictor of falls and injurious falls, whereas urinary incontinence measured by lower urinary tract symptoms (LUTS) increases the risk of falls and injurious falls. Nevertheless, the development of prospective analysis within the SCOPE study will help to clarify the association between CKD and falls. Additionally, these findings suggest that treating LUTS and physical limitation in older CKD patients might be an appropriate way to reduce risk of falling.

In the paper by Tap et al. cognitive impairment and depressive symptoms were not more prevalent among community-dwelling older persons in more advanced stages of CKD than in those without or in earlier stages of CKD. Kidney function was comparable in those with and without any signs of cognitive or mood disorders. The identification of CKD as modifiable risk factor for cognitive impairment and depressive symptoms in late life might be relevant in order to optimize therapeutic strategies. Even in this case, longitudinal studies might give additional information on the possible effect of kidney function and/or CKD progression on mental health in late life.

Artzi-Medvedik et al. showed that CKD and its severity may be significantly associated with impaired Quality of Life (QoL) among community-dwelling older people, even after adjusting for several confounders. Additionally, physical performance and comorbidities were found to affect the association between CKD and QoL, which suggest that efforts should be made to decrease the effects of such modifiable risk factors in an attempt to improve QoL of CKD patients.

Taken together, the above studies prompt the assessment of nutritional status, sarcopenia, falls, mental health, quality of life, multimorbidity and physical function, and may be warranted in the usual care of older people with impaired kidney function, which could allow for their early detection and trigger proper interventions.

## Data Availability

Not applicable.

## References

[1] Qaseem A, Hopkins RH, Sweet DE, Starkey M, Shekelle P (2013). Clinical guidelines Committee of the American College of P: screening, monitoring, and treatment of stage 1 to 3 chronic kidney disease: a clinical practice guideline from the American College of Physicians. Ann Intern Med.

[2] Molitoris BA (2014). Screening: screening for kidney disease--a lost opportunity. Nat Rev Nephrol.

[3] Collaboration GBDCKD (2020). Global, regional, and national burden of chronic kidney disease, 1990-2017: a systematic analysis for the global burden of disease study 2017. Lancet.

[4] Komenda P, Ferguson TW, Macdonald K, Rigatto C, Koolage C, Sood MM, Tangri N (2014). Cost-effectiveness of primary screening for CKD: a systematic review. Am J Kidney Dis.

[5] Go DS, Kim SH, Park J, Ryu DR, Lee HJ, Jo MW (2019). Cost-utility analysis of the National Health Screening Program for chronic kidney disease in Korea. Nephrology (Carlton).

[6] Howard K, White S, Salkeld G, McDonald S, Craig JC, Chadban S, Cass A (2010). Cost-effectiveness of screening and optimal management for diabetes, hypertension, and chronic kidney disease: a modeled analysis. Value Health.

[7] Kondo M, Yamagata K, Hoshi SL, Saito C, Asahi K, Moriyama T, Tsuruya K, Yoshida H, Iseki K, Watanabe T (2012). Cost-effectiveness of chronic kidney disease mass screening test in Japan. Clin Exp Nephrol.

[8] Lattanzio F, Corsonello A, Abbatecola AM, Volpato S, Pedone C, Pranno L, Laino I, Garasto S, Corica F, Passarino G (2012). Relationship between renal function and physical performance in elderly hospitalized patients. Rejuvenation Res.

[9] Roshanravan B, Khatri M, Robinson-Cohen C, Levin G, Patel KV, de Boer IH, Seliger S, Ruzinski J, Himmelfarb J, Kestenbaum B (2012). A prospective study of frailty in nephrology-referred patients with CKD. Am J Kidney Dis.

[10] Fried LF, Lee JS, Shlipak M, Chertow GM, Green C, Ding J, Harris T, Newman AB (2006). Chronic kidney disease and functional limitation in older people: health, aging and body composition study. J Am Geriatr Soc.

[11] Pedone C, Corsonello A, Bandinelli S, Pizzarelli F, Ferrucci L, Incalzi RA (2012). Relationship between renal function and functional decline: role of the estimating equation. J Am Med Dir Assoc.

[12] Kurella M, Chertow GM, Fried LF, Cummings SR, Harris T, Simonsick E, Satterfield S, Ayonayon H, Yaffe K (2005). Chronic kidney disease and cognitive impairment in the elderly: the health, aging, and body composition study. J Am Soc Nephrol.

[13] Deva R, Alias MA, Colville D, Tow FK, Ooi QL, Chew S, Mohamad N, Hutchinson A, Koukouras I, Power DA (2011). Vision-threatening retinal abnormalities in chronic kidney disease stages 3 to 5. Clin J Am Soc Nephrol.

[14] Duenhas MR, Draibe SA, Avesani CM, Sesso R, Cuppari L (2003). Influence of renal function on spontaneous dietary intake and on nutritional status of chronic renal insufficiency patients. Eur J Clin Nutr.

[15] Morley JE, Abbatecola AM, Argiles JM, Baracos V, Bauer J, Bhasin S, Cederholm T, Coats AJ, Cummings SR, Evans WJ (2011). Sarcopenia with limited mobility: an international consensus. J Am Med Dir Assoc.

[16] Foley RN, Wang C, Ishani A, Collins AJ, Murray AM (2007). Kidney function and sarcopenia in the United States general population: NHANES III. Am J Nephrol.

[17] Stevens PE, Lamb EJ, Levin A. Integrating guidelines, CKD, multimorbidity, and older adults. Am J Kidney Dis. 2015;65(3):494–501. 10.1053/j.ajkd.2014.09.024. Epub 2014 Dec 4. 10.1053/j.ajkd.2014.09.024 25483849

[18] Levey AS, Inker LA, Coresh J (2014). GFR estimation: from physiology to public health. Am J Kidney Dis.

[19] Corsonello A, Tap L, Roller-Wirnsberger R, Wirnsberger G, Zoccali C, Kostka T, Guligowska A, Mattace-Raso F, Gil P, Fuentes LG (2018). Design and methodology of the screening for CKD among older patients across Europe (SCOPE) study: a multicenter cohort observational study. BMC Nephrol.

